# Thymoquinone’ potent impairment of multidrug-resistant *Staphylococcus aureus* NorA efflux pump activity

**DOI:** 10.1038/s41598-024-65991-5

**Published:** 2024-07-17

**Authors:** Adel Attia M. Ahmad, Sara Y. Abdelgalil, Tarek Khamis, Ashraf M. O. Abdelwahab, Dina Nader Atwa, Gamal A. Elmowalid

**Affiliations:** 1 Department of Microbiology, Faculty of Veterinary Medicine, Zagazig University, Zagazig, Egypt; 2 Department of Pharmacology, Faculty of Veterinary Medicine, Zagazig University, Zagazig, Egypt

**Keywords:** *Staphylococcus aureus*, Efflux pump inhibitor, Thymoquinone, Ciprofloxacin, Doxycillin, Biological techniques, Drug discovery, Microbiology, Molecular biology, Medical research

## Abstract

The drug efflux pump is a crucial mechanism implicated in resistance to multiple antimicrobials. Thymoquinone (TQ) has evidently demonstrated multiple activities, antibacterial being the most effective. Knowledge about TQ activity against multidrug-resistant *Staphylococcus aureus* is very scarce. Therefore, the present study was conducted to investigate TQ resistance modulation in ciprofloxacin (CIP) and doxycycline (DO) multidrug-resistant *S. aureus*. Forty-seven samples were collected from different sources, and *S. aureus* was isolated and identified. Then, *S. aureus* resistance profiles to antimicrobials, *N. sativa* essential oil, and TQ; the correlation between TQ-MIC readings and disc diffusion; cartwheel and ethidium bromide (EtBr) accumulation assays; and *norA* gene expression were all described within silico molecular docking for TQ interactions with norA efflux pump protein. TQ-MICs ranged from 5–320 µg/ml. TQ down-regulated *norA* gene expression, resulting in a drop in efflux pump activity of 77.5–90.6% in the examined strains, comparable to that observed with verapamil. Exposure of *S. aureus* strains to CIP and DO raises the initial basal efflux pumping expression to 34.2 and 22.9 times, respectively. This induced efflux pumping overexpression was substantially reduced by 97.7% when TQ was combined with CIP or DO. There was a significant reduction of MICs of CIP and DO MICs by 2–15 and 2–4 folds, respectively, after treatment with 0.5XMIC-TQ in resistance modulation assays. These results refer to TQ ligand inhibitory interactions with NorA protein in molecular docking. Interpretations of inhibition zone diameters (IZDs) of disc diffusion and TQ-MICs exhibit independence of MICs from IZDs, as indicated by invalid linear regression analysis. TQ significantly reduced efflux pumping *S. aureus* induced by CIP and DO, but further investigations are needed to improve TQ-pharmacokinetics to restore CIP and DO activity and suppress fluoroquinolone and doxycycline-resistant *S. aureus* selection in clinical and animal settings.

## Introduction

Extremely antibiotic-resistant *Staphylococcus aureus* (*S. aureus*) is widely spread in developing countries. Most of these isolates depend on the MDR efflux system (MES), which is implicated in resistance to multiple antimicrobials. To overcome the extrusion of antibiotics out of bacterial cells, an alternative combination of the antibiotics used with natural MES inhibitors is currently expanding drastically^1^. Efflux systems comprise a single-class extruding system that induces resistance to multiple classes of antibiotics. Overexpression of MDR efflux pump genes on a specific substrate promotes resistance to other classes of antibiotics and reduces susceptibility to biocides^2^.

Efflux pump inhibitors (EPIs) aim to accumulate the antibiotic of choice intracellularly without raising the antibiotic administered dose, shift the exposed resistant bacteria to the sensitive category, and reduce the development of bacterial resistance. Efflux activity was found to be completely reversed by the efflux inhibitors, e.g., reserpine^3^ Potential efflux inhibitor screening was examined in one of the following ways: (i) minimum inhibitory concentrations (MIC) reduction of an antibiotic by sub-inhibitory concentration of a crude extract in a checkerboard synergy assay^4^. (ii) Direct measurement of fluorescent dye extrusion out of bacterial cells^5^. (iii) Testing for increased activity of an antibiotic in the presence of these EPIs, e.g., reserpine (reserpine assay). (iv) Measurement of accumulated drug inside the bacterial cell by liquid chromatography or mass spectrometry-based assay^6^. (v) Rapid demonstration of EtBr efflux pump activity in bacteria by agar-based methods^7^. (vi) In silico docking testing for identification of interactions between the EPIs and their targets, followed by in-vitro validation of MIC determination (checkerboard synergy assay) or fluorescence-based ethidium accumulation assay^8^. (vii) Detection of modulated efflux pump genes by the activities of inhibitory compounds by real-time PCR^9^. Plasmids and chromosomes in *S. aureus* encode more than 15 types of efflux pumps. Chromosomally expressed efflux proteins NorA, NorB, NorC, MdeA, and SdrM are members of the major facilitator superfamily (MFS). These members, in addition to MepA (a member of the Multidrug and Toxic Compound Extrusion family, MATE), are responsible for multidrug resistance to macrolides, tetracyclines, and fluoroquinolones and toxic extrusion^10^. The NorA is considered the predominant efflux pump via the proton motive force^11^ and is overexpressed in 43% of *S. aureus* strains^12^. Attempts were undertaken to discover and identify efflux inhibitors that exhibit comparable potency to reserpine. Compounds with efflux pump inhibition, such as dihydroergotamine, pimozide, ergoloid, and naftifine, along with others like azelastine and nilotinib, were found to have even greater efficacy than reserpine. However, it’s worth mentioning that several natural and synthetic EPIs may unfortunately lack clinical approval and/or achieve insufficient plasma levels^13^. The U.S. Food and Drug Administration (FDA)-approved drugs, including diclofenac sodium, domperidone and glyceryl trinitrate, verapamil, and metformin, were examined as safe potential EPIs to combat the antibiotic resistance in *S. aureus*^14^. Metal nanoparticles (copper, silver, and zinc oxide) act as a competitive inhibitor of antibiotics for the binding site of efflux pumps or disrupt membrane potential^15^, leading to a reduction of bacterial resistance through blocking the extrusion of antibiotics outside the bacterial cell. Additionally, they block the efflux of quorum-sensing molecules and, hence, hinder bacterial biofilm formation^16^.

Natural bioactive polyacylated oligo-saccharides have antibacterial activity and induce efflux of norfloxacin in the MDR *S. aureus* strain^17^. Essential oils from multiple edible plants contain chemical compounds that represent safe EPIs^18^. However, phytochemicals extracted from *Artemisia absinthium* were considered anti-biofilm agents and inhibited MFS pumps in *Escherichia coli* and *Enterococcus faecalis*^19^. Extracts of a medicinal plant (*Myristica fragrans*) were proven to enhance the efficacy of ciprofloxacin by inhibiting 80% of MRSA growth with expression of the *norA* and *mepA genes*^20^. Exposure to ciprofloxacin (CIP) prompts resistance evolution in certain lineages of *S. aureus*. Accelerated evolution of resistance to CIP was observed with elevated levels of *norA* expression, and it is known to contribute to the intrinsic ciprofloxacin resistance in *S. aureus*^21^. The molecular mechanisms underlying the susceptibility of *S. aureus* to *N. sativa* are still not fully understood. In the present work, resistance modulation to fluoroquinolone and doxycycline in *S. aureus* by TQ, *norA* gene regulation, and TQ-MIC prediction according to IZDs of disc diffusion were reported.

## Materials and methods

### Bacterial isolation and identification

To isolate and identify *S. aureus* strains in the study, a total of 47 samples were collected from different sources. The samples were collected from cows’ milk (MK) (n = 25; chronic mastitis); minced beef meat (MM) specimens (n = 15) from diverse markets in Zagazig city, Sharqiah governorate, Egypt; urine (UR) (n = 5) and pus (P) (n = 2) samples of human subjects were obtained from private clinical bacteriology laboratories. All samples were collected in December 2022. A loopful of broth culture was inoculated onto mannitol salt agar. Suspected colonies were inoculated into brain heart infusion and incubated at 37 ℃ for 24 h. Developed colonies were characterized through 16S rRNA and they were examined for coagulation of sheep plasma in tube coagulase test as described^22^. One colony of pure culture was examined for qualitative detection of both clumping factor and protein A using Blue Staph Latex Kits (Pro-Lab Diagnostics) according to the instructions of the manufacturer. Coagulase-positive strains were preserved in 10% glycerol at − 80 ℃ until used.

### *Nigella sativa* essential oil and thymoquinone

*Nigella sativa* (*N. sativa*) essential oil (EO), cold-pressed extracted (20.0%) black cumin oil, was purchased from Harraz Herbal Products Company, Egypt. The EO was dissolved in 10% DMSO. Thymoquinone (TQ), 99%, was purchased from Sigma (274666-5G, Sigma, Sant Louis, MO, USA). A total of 250 mg of TQ were dissolved in 10% DMSO and preserved as a stock solution at − 20 ℃. The stock solution was diluted in 1.0 ml of 1:1 ethanol/H_2_O and used in appropriate concentration.

### Disc diffusion

#### Susceptibility to antimicrobials

The identified *S. aureus* strains were examined for susceptibility testing to antimicrobials as previously done^23^ using the following antibiotic discs: amoxicillin (AM), oxacillin (OX), amoxycillin/clavulanic acid (AMC), ampicillin/sulbactam (SAM), cefepime (CPM), linezolid (LZD), doxycycline (DO), trimethoprim/sulpha-methoxothazole (SXT), chloram-phenicol (C), azithromycin (AZM), gentamicin (CN), ciprofloxacin (CIP), and clindamycin. Briefly, antibiotic discs were fixed onto Muller Hinton agar (MHA) previously swabbed with 0.5 MacFarland bacterial density suspension of freshly isolated colonies. The inhibition zone diameters (IZDs) were recoded after incubation for 24 h. at 37 ℃. The obtained data were interpreted according to the Clinical and Laboratory Standards Institute guidelines (CLSI, 2020)^24^.

#### Susceptibility to *N. sativa* essential oil and thymoquinone

The antibacterial susceptibility of *S. aureus* strains to *N. sativa* EO and TQ was examined on MHA supported with 1% glucose following the established procedure^25^. Briefly, for the susceptibility testing of *S. aureus* strains to *N. sativa* EO, MHA culture plates were swabbed with 0.5 MacFarland bacterial density suspension. Sterile 6 mm sterile Whatman filter paper No. 1 discs were fixed onto agar medium and then loaded with 20 µl EO (20 mg dry matter) dissolved in 10% DMSO. For the susceptibility of *S. aureus* strains to TQ, Whatman filter paper discs were fixed onto the agar medium and then loaded with 20 µl (50 µg TQ) dissolved in 10% DMSO and alcohol. Control discs with 10% DMSO were included. Culture plates were incubated for 24 h at 37 °C, and then the IZDs were recorded in millimeters.

### Broth microdilution of *N. sativa* essential oil and thymoquinone

The minimum inhibitory concentrations (MIC) of *N. sativa* EO and TQ were determined by growing bacteria using the broth microdilution method in 96-well microtiter plates according to the guidelines described by the CLSI. Briefly, *N. sativa* oil and TQ were serially double-fold diluted in 100 µl MH broth. One hundred µl of MH broth containing 2 × 10^5^ logarithmic-phase cells was added to each well. Plates were covered with adhesive tape and incubated for 24 h at 37 °C.The last non-turbid well was recorded as MIC.

### Broth microdilution of ciprofloxacin and doxycycline combined with thymoquinone

The MICs of CIP and doxycillin (DO) were estimated to be parallel to those of combinations of CIP + TQ and DO + TQ against the examined strains. The MIC change of the drug after the addition 0.5 MIC of TQ was recorded as done^26^. Briefly, the drug was serially double-fold diluted in 100 µl BHI in sterile 96-well microtiter plates, and 100 µl BHI containing 2 × 10^5^ logarithmic-phase cells were added to each well. For drug combination with TQ, the drug was serially double-fold diluted in 100 µl brain heart infusion in sterile 96-well microtiter plates, and 100 µl containing 2 × 10^5^ logarithmic-phase cells were added with 0.5 MIC of TQ per well. Similarly, the same test was run with the addition of verapamil (VP, 200 mg/l) as an efflux pump inhibitor and benzalkonium chloride (BZK, 1.0 mg/l) as an efflux pump inducer. The plates were sealed with adhesive tab and then incubated at 37 ℃ for 24 h. At the end of incubation, MICs data were analyzed using contemporary breakpoint criteria of DO susceptibility at ≤ 4 µg/ml. and resistance at ≥ 16 µg/ml. CIP susceptibility is at ≤ 1 µg/ml and resistance is at ≥ 4 µg/ml. MIC changes of CIP + TQ, CIP + VP, DO + TQ, and DO + BZK were recorded.

### Thymoquinone synergy with ciprofloxacin or doxycycline onto agar surface

The synergism between TQ and CIP or DO was investigated following the method outlined by^27^. Briefly, MHA was seeded with 0.5 MacFarland bacterial density suspension. Sterile 6 mm Whatman filter paper No. 1 discs were fixed onto agar surface and loaded with 20 µl (25 µg) of TQ. The plates were incubated at room temperature for 15 min. Filter paper discs were removed, and CIP (ofloxacin; one of the quinolones was used) or DO discs were fixed on the same site. CIP and DO control discs were included. Plates were incubated for 24 h at 37 °C. IZDs of both the test and the control were recorded.

### Efflux pump estimation in agar (cartwheel method)

The assessment of EtBr efflux by *S. aureus* was achieved by growing the strains onto BHI agar with varying concentrations of EtBr using the EtBr-cartwheel (EtBrCW) agar method as described^7^. Briefly, freshly prepared BHI agar was poured in two sets with varying concentrations of EtBr (0.0–2.5 mg/l). Plates with varying EtBr concentrations were inoculated with freshly isolated strains (10^7^ CFU/ml) in a cartwheel pattern (eight isolates in a radial manner per plate). Positive and negative strains for EtBr efflux pumping were included in each culture plate. Plates that had been incubated overnight at 37 ℃ were observed under a UV transilluminator. Strains that exhibited fluorescence and those without fluorescence were subjected to a retest using the EtBrCW. This retesting was conducted after treatment with 0.5 MIC of TQ, which is an efflux inhibitor, and benzalkonium (BZK) as an efflux inducer on the medium surface.

### Ethidium bromide accumulation assay

Anti-efflux activity in *S. aureus* strains treated with TQ and verapamil was determined in the EtBr accumulation assay as described^28^. Briefly, 100 µl BHI containing 1% glucose, 10^6^ CFU, and 0.5 MIC-TQ were dispensed in black microtiter plates and incubated for 15 min at 37 ℃ with EtBr (10 µg/ml). The same test was conducted using VP (200 mg/l) as a positive control. The kinetics of intracellular EtBr accumulation were measured at 490 nm excitation and 579 nm emission using a Synergy HT reader (Biotech) after 15, 30, and 45 min. The mean of three readings was recorded. The binding of EtBr to the DNA of *S. aureus* was related to the increased fluorescence intensity recorded by the reader, while the extrusion of EtBr was related to decreased fluorescence over time.

### Quantitative real time PCR (qRT-PCR)

The *S. aureus* strains exhibiting variable susceptibility to CIP and DO were investigated for regulation of the *norA* efflux pump gene. Gene expression of non-treated and treated strains with sub-MICs of DO, CIP, TQ, and combination regimens was assessed. *S. aureus* strains (codes MK8, MK4, UR3 and MM1, Table 3) were grown in Mueller Hinton broth (without antibiotics). Total RNA was extracted using the RNeasy^®^ mini kit (Qiagen, Germany) according to the manufacturer’s instructions. Total RNA concentration was measured using a QuaWell UV–VIS spectrophotometer (Quawell Technology, Inc., USA). The purity of the RNA from proteins and salts was checked by calculating the 260/280 ratio. The accepted range fell in the area of 1.8–2. In addition, the 230/260 ratio was assayed to check the further purity of the RNA, in which the accepted value ranged from 2.0–2.2. cDNA was synthesized from 500 ng of the total extracted RNA with a high-capacity cDNA reverse transcriptase (Applied USA). The relative expression of the *norA* gene was done with Rotor Gene Q 2plex (Qiagen, Germany) with a total reaction volume of 20 µL (10 µL Top Real Syber Green master mix (Enzynomics, Korea) and 1 µL of the forward and reverse predesigned specific primers (Sangon Biotech, Beijing, China). *NorA* F-TTGCTATTACGGGTGGCGGT, R-TCAATCCGCCTGCAAAGCCT, (270 bp)^29^, 16S F: GGACGGGTGAGTAATGTC and R: TCTCAGACCAGCTAGGGATCG, (193 bp)^30^, and nuclease-free water up to 20 µL) with a cycling condition of initial DNA denaturation at 95 ℃for 10 min, then 40 cycles of denaturation at 95 ℃for 10 s, annealing at 60 ℃ for 15 s, and extension at 72 ℃ for 20 s, followed by a melting curve analysis. The relative expression of the *norA* efflux pump gene was determined using the ΔΔCt method^31^. The data was normalized using 16S as a housekeeping gene according to its stable expression among different treatments that was checked with GeNorm.

### Molecular docking

The NorA protein 3D crystal structure was obtained from the protein data bank (PDB): https://www.rcsb.org/structure/8DYS. Autodock MGL tools were used for the preparation of the macromolecule, ligand, and grid box dimensions. The TQ-norA docking process was done via autodock Vina 1.2.0 software^32^ and the visualization results were performed using BIOVIA Discovery Visualization 2024 client software.

### Statistical analysis

Linear regression analysis was used to predict zone diameter values (dependent variables) of *N. sativa* EO and TQ based on the value of MIC (independent variable) by reference broth microdilution. The prediction was represented by a simple linear regression.

## Results

### Antimicrobial susceptibility to antibiotics and *N. sativa*

**Figure 1:**
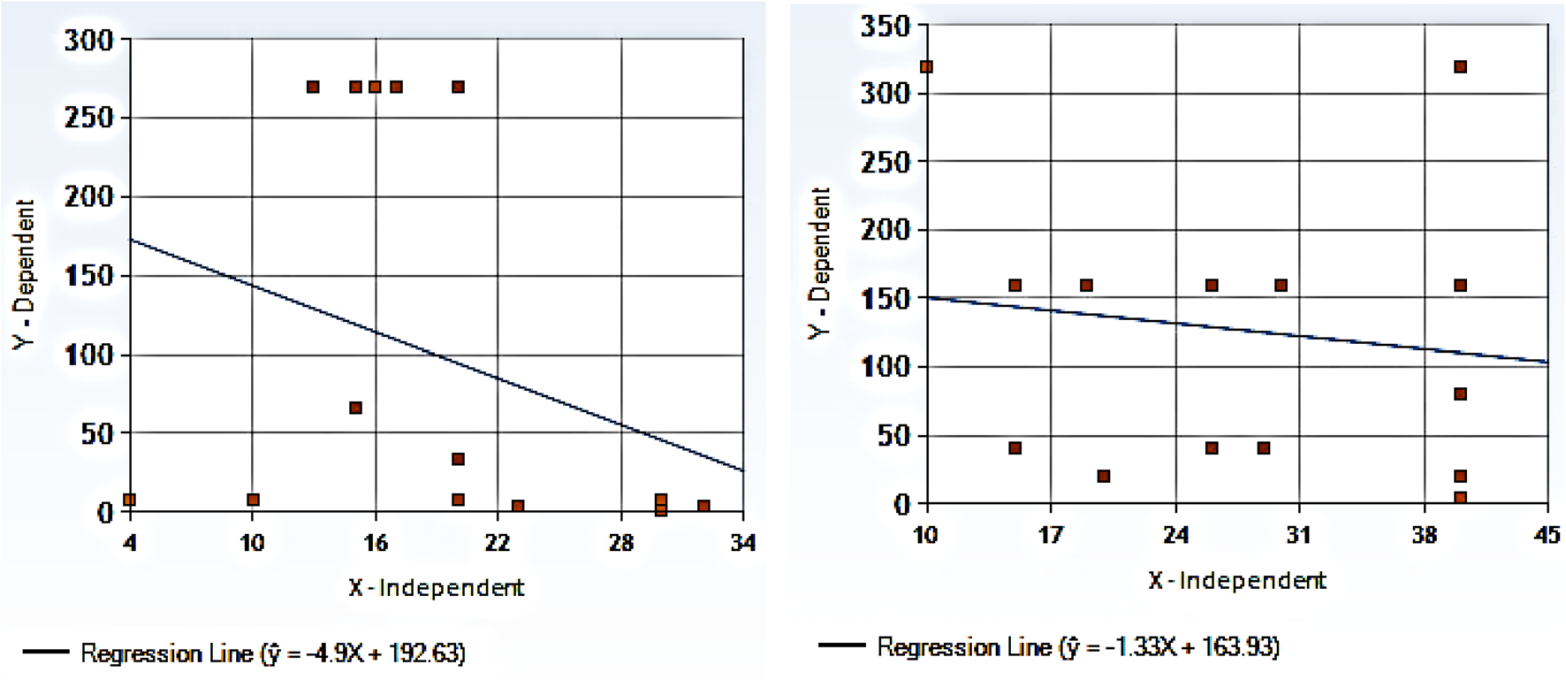
(**A**,**B**) Regression lines of *N. sativa* essential oil (**A**, left) and thymoquinone (**B**, right) of *S. aureus* strains. The test was carried on 18 *Staphylococcus aureus* strains and the inhibition zone diameters (X-axis) obtained with 20 mg *N. sativa* dry matter/disc and 50 µg thymoquinone/disc were plotted. Invalid regression lines showed lack of correlation between inhibition zone diameters and MICs (Y axis) of *N. sativa* essential oil or thymoquinone.

Linear regression analysis was used to predict zone diameter values (dependent variables) of *N. sativa* EO and TQ based on the value of MIC (independent variable) by reference broth microdilution. The prediction was represented by a simple linear regression (Fig. 1).

### Independence of *N. sativa* MICs from disc inhibition zone diameters

The agreement between the commonly used simple disc diffusion test for evaluation of *N. sativa* EO and TQ was investigated in comparison with the reference broth microdilution (BMD) method outlined by the Clinical and Laboratory Standards Institute (CLSI) (Table 1, Fig. 1). IZDs of disc diffusion and MICs of *N. sativa* EO and TQ by broth microdilution against *S. aureus* strains obtained from different sources are shown in Table 1. There was no observed correlation between the MIC readings of EO and TQ and the corresponding IZDs for the same strains when using 20 mg dry matter/disc and 50 µg/disc, respectively. The MIC results of disc diffusion could not be categorized in relation to IZDs. Six resistant strains to TQ with high MICs ranging from 160–320 µg/ml showed IZDs ranging from 20–40 mm by disc diffusion. This suggests poor growth of TQ-resistant strains onto MHA medium (Table 1). Similarly, four strains resistant to *N. sativa* EO with high MICs ranging from 66–269 mg dry matter/ml showed IZDs of the same strains ranging from 15–20 mm by disc diffusion, indicating poor growth of *N. sativa* EO-resistant strains onto MHA medium.

**Table 1:** Susceptibility of *Staphylococcus aureus* strains from different sources to antimicrobials.

Strain origin code	Antimicrobials												EO	TQ
	SAM	AMC	OX	FEP	CIP	CN	C	LNZ	DO	SXT	DA	AZM	IZD**▫**/MIC (µg/ml)	IZD●/MIC (µg/ml)
MK1			x	x	x				x	x	x	x	17/269	26/160
MK2	x	x	x	x	x				x	x	x	x	17/269	19/160
MK3			x		x					x	x	x	20/269	26/40
MK4	x	x		x		x	x	x		x	x		20/8	40/320
MK5	x	x	x	x									15/66	10/320
MK6	x	x	x	x							x	x	24/8	40/5
MK7	x	x		x	x	x				x	x	x	20/34	29/40
MK8	x			x	x							x	23/4	20/20
MM1			x	x	x		x	x	x	x	x	x	13/269	40/80
MM2	x	x		x		x	x	x	x	x	x	x	15/269	15/160
MM3	x	x		x	x			x		x		x	10/8	40/160
UR1	x	x	x	x	x	x	x	x	x	x	x	x	25/65	15/160
UR2	x	x	x	x	x	x	x	x		x	x	x	16/269	30/160
UR3	x	x	x	x	x	x			x	x	x	x	32/4	15/40
UR4	x		x	x		x				x	x	x	30/8	40/20
UR5		x					x			x	x	x	30/8	40/80
PS1	x	x	x	x	x	x		x	x	x	x	x	30/1	40/320
PS2	x			x			x	x	x	x	x	x	20/8	40/5

Empty boxes and boxes with x refer to sensitive and resistant category, respectively.

*EO N. sativa* essential oil, *TQ* thymoquinone, *SXT* trimethoprim/sulphamethoxazol, *CIP* ciprofloxacin, *C* chloramphenicol, *DO* doxycycline, *LZD* linezolid, *AZM* azithromycin, *CN* gentamicin, *OX* Oxacillin, *FEP* cefepime, *DA* clindamycin, *AMC* amoxycillin/clavulanic acid, *SAM* ampicillin/sulbactam. *MK* milk, *MM* minced meat, *UR* urine, *PS* pus, *IZD* inhibition zone diameter (mm), *TQ* thymoquinone *●* IZD (50 µg/disc), *◦* IZD (20 mg dry matter/disc).

Linear regression analysis of IZDs produced by 20 mg/disc and MICs of *N. sativa* EO was conducted (Fig. 1A). Similarly, IZDs produced by 50 µg of TQ/disc were plotted against TQ-MICs (Fig. 1B). Scalar readings of IZDs showed no linear correlation with the MIC readings of CLSI broth microdilution (BMD). Linear regression analysis revealed that unknown MIC values could not be predicted from the estimated zone diameters. The linear regression analysis of IZDs and corresponding MICs of both *N. sativa* EO and TQ was invalid.

### Resistance modulation of ciprofloxacin and doxycycline in broth microdilution

The activity of CIP and DO was evaluated in the presence and absence of sub-inhibitory concentrations of TQ against *S. aureus* (Table 2). Eight out of ten (80%) strains showed CIP-MICs drop by 2–15 folds after combination with 0.5 MIC of TQ, while 60% of strains showed DO-MICs drop by 2–4 folds. TQ reduced MICs in both the sensitive or resistant categories of CIP and DO. Verapamil was weaker than TQ to reduce CIP-MICs in the resistance modulation assay. In the presence of TQ, two strains remained resistant to CIP, and one strain remained resistant to DO.

**Table 2:** Accumulation and efflux of ethidium bromide by the cartwheel method; minimal inhibitory concentration of TQ, CIP, DO, and combination regimen in resistance modulation assay.

Strain code origin	Cartwheel			MIC (µg/ml) of CIP, DO and combination regimen						
	Test	Control								
		TQ	BZK	TQ	CIP	CIP+TQ	CIP+VP	DO	DO+TQ	DO+BZK
MK8	NF	F	NF	20	(R) 25	(S) 15↓	NC	(S) 3	4↓	3↑
MK4				320	(S) 1.5	15↓	NC	(S) 3	4↓	NC
MM1				80	(S) 0.40	4↓	3↓	(R) 60	(S) 5↓	2↑
PS2				5	(S) 3	3↓	4↓	(S) 1.5	3↓	4↑
UR5	F			80	(S) 1.6	4↓	1↓	(S) 15	3↓	3↑
UR4				20	(S) 1.5	2↓	NC	(R) 16	(S) 2↓	1↑
MK6				5	(S) 1.5	4↓	3↓	(S) 0.7	NC	NC
MK5				320	(R) 12	(R) 2↓	(R) 1↓	(S) 0.18	NC	NC
UR3				40	(R) 30	NC	2↓	(R) 120	NC	NC
MK1				160	(S) 3	NC	NC	(S) 0.18	NC	NC

↑: refer to MIC fold increase, ↓: refer to MIC fold decrease.

*NC* no change, *TQ* thymoquinone, *VP* verapamil, *BZK* benzalkonium chloride, *R* resistant, *NC* no change, *(S)* sensitive, *MM* minced meat, *UR* urine, *PS* pus, *MK* milk, *CIP* ciprofloxacin, *DO* doxycycline.

### Resistance modulation of CIP and DO onto the agar surface

**Figure 2:**
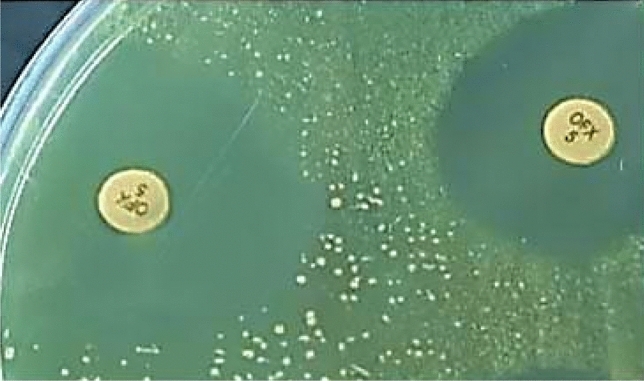
Agar disc diffusion of ofloxacin (OFX5, right) and thymoquinone/ofloxacin synergism (TQ/OFX5, left) against *Staphylococcus aureus* strain. The ofloxacin disc was fixed after the removal of the thymoquinone (TQ) disc (25 µg). The TQ/OFX5 synergy showed a wider inhibition zone. The strain has become more sensitive to OFX with non-entire zone boundaries dispersing bacterial seed inoculum (left). This strain showed a TQ-MIC drop in the resistance modulation assay.

To validate the resistance modulation of CIP and DO by TQ observed in broth microdilution, a parallel resistance modulation assay of CIP (ofloxacin disc was used instead of CIP disc) or DO by TQ was examined on the MHA surface (Fig. 2). Resistant strains responded to resistance modulation in broth microdilution; these strains reverted to the sensitive category on the agar after combination with TQ. Moreover, the sensitive strains to CIP and DO exhibit wider inhibition zones in comparison with the control.

### Qualitative and quantitative uptake and efflux of ethidium bromide

**Figure 3:**
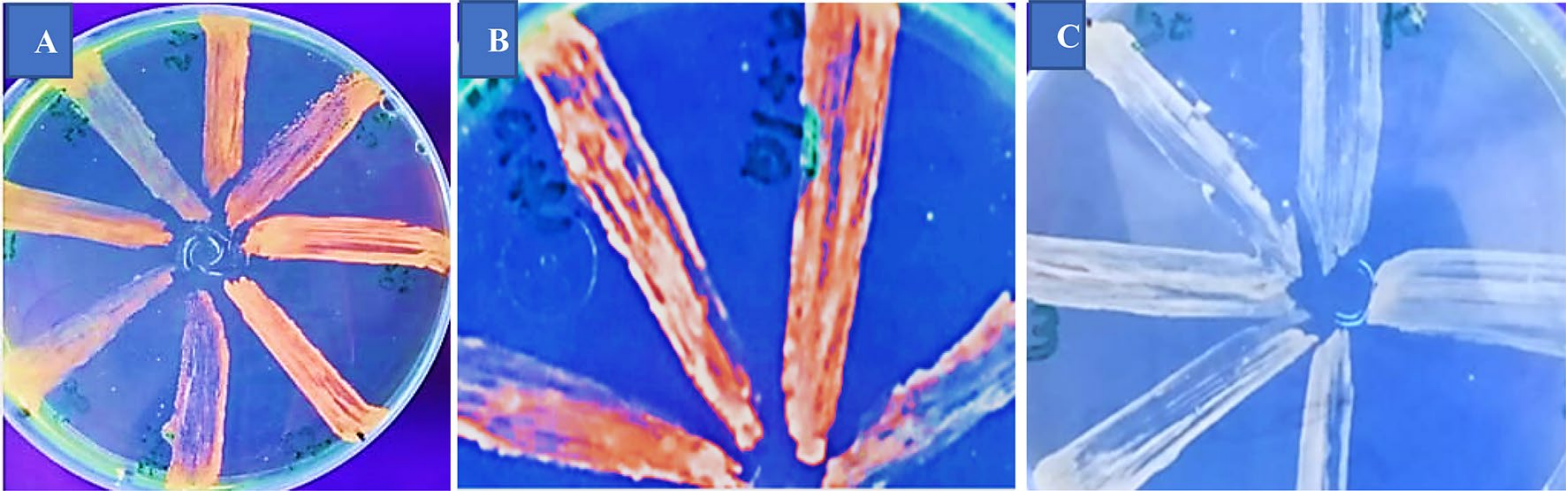
The cartwheel method results onto Muller-Hinton agar (0.5 mg/L ethidium bromide, EtBr). (**A**) shows all the *Staphylococcus aureus* streaks with fluorescence (−ve efflux). (**B**) shows *S. aureus* strains (ID and MM1) on the left side without fluorescence (+ve efflux); the same strains show fluorescence (−ve efflux) after treatment with verapamil, thymoquinone, and verapamil (EtBr efflux inhibitors), respectively, in the following streaks. FC shows that *S. aureus* streaks lost fluorescence after treatment with benzalkonium chloride (an efflux inducer).

**Figure 4:**
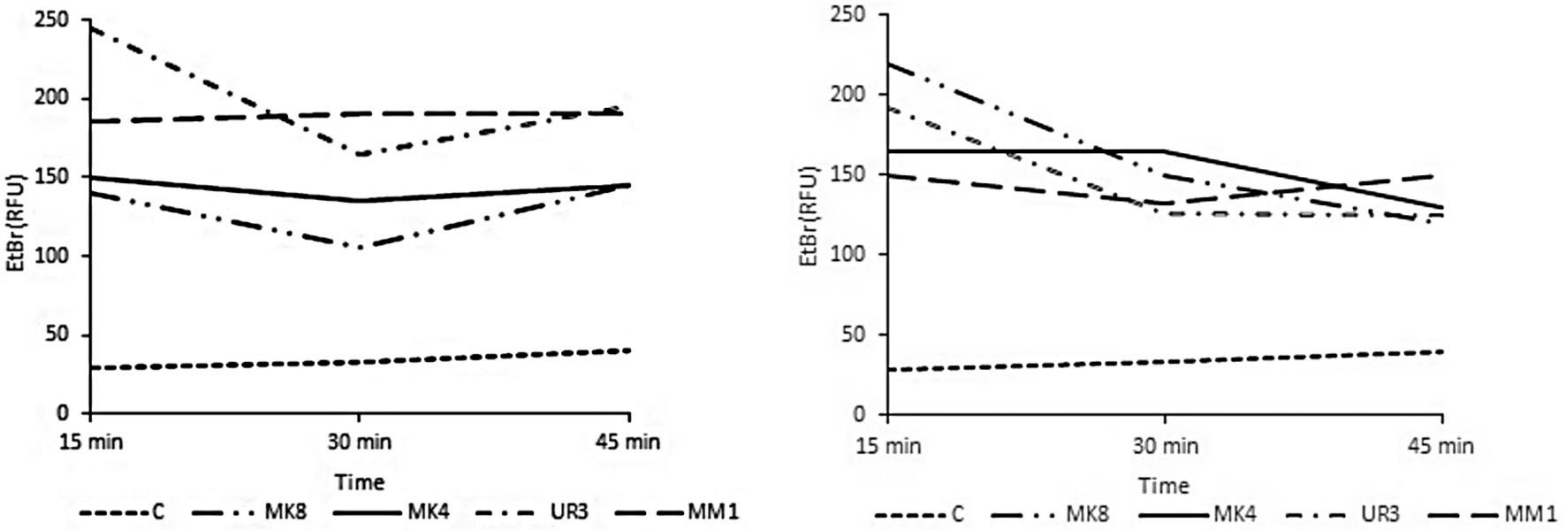
Quantitative detection of EtBr uptake and efflux in four *Staphylococcus aureus* strains (MK8, MK4, UR3, and MM1) treated with thymoquinone (left) and verapamil (right) and mean readings of control non-treated strains (C). Ethidium bromide (EtBr) accumulation in *Staphylococcus aureus* strains (codes, MK8, MK4, UR3, and MM1) after treatment with thymoquinone (left fig.) showed EtBr uptake at 105-245 RFU fluorescence. After treatment of these strains with verapamil (positive control, right fig.), they showed EtBr uptake at 125-220 RFU fluorescence, referring to EtBr efflux inhibition. Meanwhile, control non-treated stains (--C) showed potent efflux pumping at reduced fluorescence (29–40 RFU).

Cartwheel method results showed the qualitative effect of ethidium bromide (0.5 mg EtBr) inoculation on MHA-cultured *S. aureus* strains (Fig. 3). Qualitative and quantitative uptake and efflux of EtBr in *S. aureus* strains were evaluated by EtBrCW (0.5 mg EtBr/l culture medium, Table 2, Fig. 4). Except for four strains, all strains showed EtBr accumulation in EtBrCW (Table 2). These strains in the EtBr accumulation assay showed EtBr uptake and efflux (positive strains for efflux pumping, 29–40 relative fluorescence units (RFU). Upon treatment with TQ, these strains exhibited fluorescence levels of 105-245 RFU, indicating potent inhibition of EtBr efflux (Fig. 4). TQ and VP showed comparable levels of EtBr accumulation, and they maintained stable levels of fluorescence up to 45 min. in the EtBr accumulation assay, where the fluorescence is inversely related to the strain capacity to extrude the substrate. Thymoquinone and verapamil-treated strains showed 105-245 and 125-220 RFU fluoresence, respectively, referring to EtBr efflux inhibition. Control strains showed potent efflux pumping with 29–40 RFU fluorescence (Fig. 4).

### *norA* gene regulation

The relative expression of the *norA* gene was assessed after bacterial exposure to sub-MICs of TQ, CIP, DO, and their combination regimens (Table 3). The clinically isolated strains have an initial basal level (ranging from 0.977 to 1.03) of efflux pump activity, and upon their exposure to CIP and DO, this initial basal efflux pump level increased 31.6–34.2 and 19.3–22.9 times, respectively, referring to high “basal” versus “increased” levels (Table 3). In contrast, strains treated with CIP + TQ and DO + TQ displayed a 93.4–97.7% and 88.8–95.6% reduction in efflux pump activity, as evidenced by *nor*A gene downregulation. TQ downregulated the *norA* gene, leading to a 77.5–90.6% reduction in efflux pumping activity compared to non-treated strains. Results showed that TQ and VP have comparable anti-efflux pump activity; they reduced 93.4–93.6% of efflux pumping, respectively.

**Table 3:** Relative gene expression (fold-change) of the *norA* efflux gene in treated and non-treated *Staphylococcus aureus* strains.

Strains ID	Non treated	Treatment					
		TQ↓	CIP↑	CIP+TQ↓	CIP+VP↓	DO↑	DO+TQ↓
MK8	0.999	0.225 (77.5%)	34.207 (34.2)	2.253 (93.4%)	2.174 (93.6%)	22.605 (22.6)	2.350 (89.6%)
MK4	0.995	0.126 (87.4%)	34.100 (34.1)	2.297 (93.2%)	2.794 (91.8%)	22.877 (22.9)	2.121 (95.6%)
UR3	0.977	0.092 (90.6%)	30.969 (31.6)	3.328 (97.7%)	2.160 (91.0%)	21.980 (22.5)	2.050 (90.6%)
MM1	1.03	0.134 (78.0%)	35.100 (34.1)	3.400 (90.3)	2.180 (93.7%)	19.908 (19.3)	2.219 (88.8%)

(%): A drop in *norA* gene expression fold-change is related to the initial basal fold-change of non-treated strains. Numbers between parenthesis: *norA* gene overexpression fold change related to initial basal fold change of non-treated strains. ↓ refers to *norA* downregulation. ↑: refer to *norA* upregulation.

*TQ* thymoquinone, *VP* verapamil, *CIP* ciprofloxacin, *MK* milk, *MM* minced, *UR* urine.

### Thymoquinone-NorA inhibitory docking

**Figure 5:**
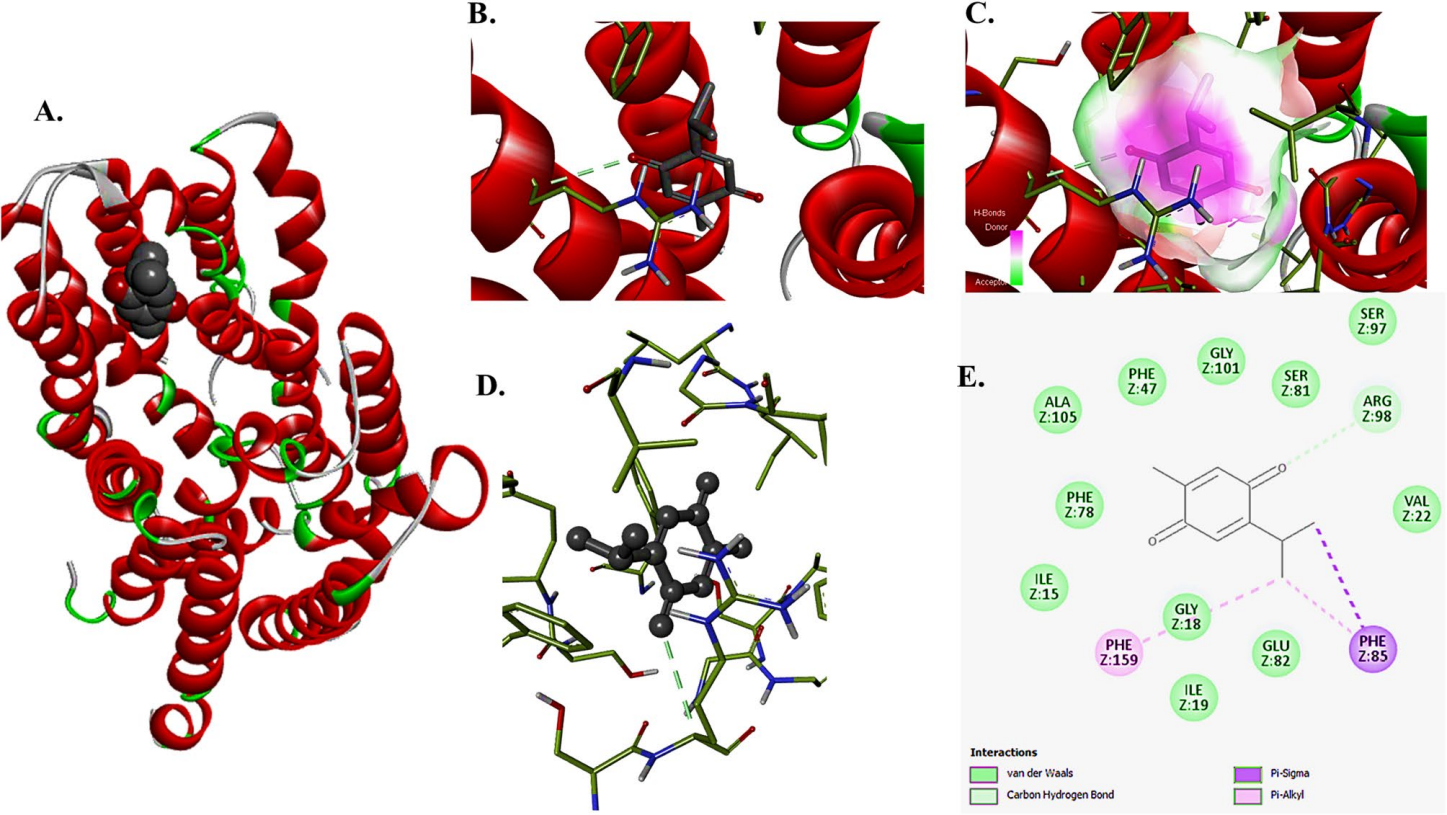
Docking of thymoquinone (TQ) ligand with efflux pump NorA protein (**A**–**E**). (**A**) 3D image showing binding of TQ with NorA protein with an affinity score of 6.9 kcal/mol, (**B**,**C**). 3D images show the binding pocket of TQ configuring the sites for hydrogen bonds. (**D**) 3D image showing the interacting atoms of the norA with the TQ. (**E**) 2D image showing different types of bonds that took place between TQ and norA.

To detect the inhibitory activity of the TQ for the efflux pump protein norA*,* a macromolecule-ligand interaction in silico study was performed. The results revealed that TQ could bind and block the activity of the NorA efflux pump with a binding efficiency score of 6.9 kcal/mol. This binding took place via van der Waals attraction force, pisigma interaction at PHE (Z:85), carbon hydrogen bond at ARG (Z:98), and pi-alkyl interaction at PHE (Z:159 and Z:85) (Fig. 5).

## Discussion

Community-acquired *S. aureus* is a major pathogen responsible for skin and soft tissue infections^33^, as well as invasive infections. Bacterial efflux systems mediate resistance to disinfectants, dyes, and detergents. The progress of bacterial resistance can be retarded by the discovery of new antibiotics or by restoring the activity of the currently used antibiotics^34^. Natural components in *N. sativa* seeds have been reported, including bactericidal and antifungal activities, as well as antihypertensive and antitumor activities^35^,^36^. *N. sativa* oil has gained attention for its antibacterial activity against Gram positive and Gram negative bacteria, and as an immunomodulator against fungi^28^,^37^,^38^. The current study aims to address three key aspects, providing insights into: (i) the relationship between IZDs obtained through disc diffusion and MICs of both *N. sativa* EO and TQ; (ii) the impact of TQ on efflux pump activity in comparison with the benchmark efflux pump inhibitor (EPI), verapamil; and (iii) the regulation of the *nor*A gene in the absence and presence of TQ. To the best of our knowledge, this is the first study to achieve MIC readings of *N. sativa* EO and TQ in correlation with the corresponding IZDs as well as *norA* gene expression under TQ effects.

Previously, EtBr efflux, resistance modulation in broth microdilution, and *mepA* gene expression revealed *N. sativa* anti-efflux pump activity^38^,^28^. The current study aimed to address three key aspects and provide insights into: (i) the prediction of TQ-MIC according to IZDs of disc diffusion; (ii) the impact of TQ on efflux pump activity in comparison with that of the benchmark efflux pump inhibitor (EPI), verapamil; and (iii) the regulation of the *norA* gene in the absence and presence of TQ. To the best of our knowledge, this is the first study to achieve MIC readings of *N. sativa* EO and TQ in correlation with the corresponding IZDs as well as *norA* gene expression under TQ effects. The results reported that TQ is responsible for the extreme reduction of *norA* gene, and resistance modulation to fluoroquinolone and doxycycline in *S. aureus*. Resistance percentages of *S. aureus* to antibiotics in dairy farms in Egypt were rising, for example, in CIP (16.0%), DO (26%), clindamycin (28%), CN (24%), C (24%), and SXT (26%), respectively, in 2020^38^ to 62.5%, 25%, 75%, 25%, 12.5%, and 62.5%, respectively. In the present study, the observed reduced resistance to chloramphenicol might be due to the restricted use as mandated by regulatory authorities.

The successful capacity of TQ as an EPI allows it to be a desirable approach for treating a variety of efflux-producing bacteria, including clinical isolates of Staphylococci, Enterococci, and Streptococci^36^. In the present study, the efficacy of DO and CIP (as two antibiotics from different antibiotic groups) against the clinical strains of *S. aureus* was successfully restored; two strains out of 10 that were resistant to DO were moved to the sensitive category by 0.5 MIC-TQ. Similarly, it was found that three strains out of eight that were resistant to tetracycline were similarly dramatically sensitive to DO.

TQ’s functional anti-efflux pump activity, expressed as the antibiotic’s MIC-drop in the resistance modulation experiment, served as a quantitative measure of anti-efflux pump activity and permitted strains grouping. On the other hand, the tested strains could not be distinguished using the EtBr accumulation assay. Tetracycline uptake and tetracycline efflux activities may not have responded to the elevated tetracycline concentration in a manner consistent with each other, according to a study by^39^. TQ has direct antibacterial effects on bacterial cell envelopes in strains with TQ-MIC below the pharmacokinetic blood maximum concentration (C-max, 3.48 µg/ml)^39^. However, strains exceedingly twice the TQ-MIC are not under the antibacterial effects. In the present work, MIC ranged from 5–320 µg/ml; therefore, only two out of 10 isolates were considered sensitive (5 µg/ml) in the blood stream according to TQ pharmacokinetic parameters. In a similar study^36^, reported that, in spite of the fact that most *S. aureus* strains were responsive to TQ-EPI, only one out of eight isolates was considered sensitive (4 µg/ml) in the blood stream according to TQ C-max, and the sensitivity depends on the anti-efflux of 0.5 MIC-TQ. Therefore, TQ exhibits low bioavailability, poor pharmaco-kinetics, and low accumulation in the target tissues. Improved pharmacokinetics can be achieved through capsulated nano-delivery systems. The nano-delivery system will raise the TQ blood C-max level. The development of nanostructured carriers for TQ formulation revealed proven success, as reported by^40^. A single dose of TQ (20 mg/kg) loaded into nanostructured lipid carriers (TQ-NLC) orally administered in rats achieved high bioavailability, faster absorption (1 h), and significantly high plasma concentrations with a C-max of 3348 ng/ml compared with that of unloaded TQ suspension (1160 ng/ml)^41^. High solubility and bioavailability produced higher antibacterial and antifungal activity after nano-formulation of Curcuma longa L. plant extract^42^.

Disc diffusion was extensively employed to evaluate EO and TQ’s antibacterial efficacy. It hasn’t been investigated before how MICs and agar diffusion are related. This work’s “invalid linear regression analysis of essential oil and TQ” resulted from the linear regression analysis of IZDs and MICs of both EO and TQ, which showed that unknown MIC values could not be predicted from the estimated IZDs. Similar to this, diffusion of vancomycin disc was not allowed in a study because the agar screen plates were unable to sustain the growth of resistance isolates (VISA, vancomycin intermediate resistant *S. aureus*) and failed to identify them as truly susceptible^43^. Consequently, the *S. aureus* vancomycin agar disk diffusion breakpoints were eliminated by CLSI. Strains displayed diversity when resistance modulation tests were employed, but the EtBr accumulation and EtBrCW assays were unable to differentiate between these stains. After associating with TQ, most strains displayed a drop in CIP and DO MIC. The latter stopped 75% of the EtBr from being extruded in the EtBr accumulation experiment (Fig. 3)^28^. When it came to inducing efflux activity and *norA* gene expression, CIP was more effective than DO at increasing *norA* gene expression and efflux activity^44^. According to the current study, TQ is more capable of reducing CIP-MIC than VP. TQ’s simultaneous actions on *S. aureus* cell envelopes and anti-efflux activities could be the reason for this variation in reduction. In *S. aureus* lineages exposed to ciprofloxacin, DNA topoisomerase mutations have been found; these changes result in increased levels of *norA* gene expression, which significantly enhances resistance evolvability^1^. The first basal efflux that the clinically isolated bacteria in this study displayed may have been caused by intrinsic resistance, or previous exposure to fluoroquinolone^45^ (Fig. 3). In the resistance modulation assay, a dramatic elevation (34.2 times) of the efflux pump was noted as a result of *norA* gene overexpression. TQ down-regulated the *norA* gene, which significantly decreased ciprofloxacin resistance. In the present work, TQ ligand produced inhibitory interactions with NorA protein in docking, and TQ as a bioactive extract compound represents an antibacterial-like drug^46^. The experimental investigations of TQ safety proved the TQ nano-liposomal formulation reduced toxicity and exhibited comparatively lower toxicity as compared to free TQ at the same dose in treated mice^47^.

## Conclusion

Thymoquinone exhibited high minimum inhibitory concentrations (MIC) of antibacterial activity. The detection of TQ-MIC against *Staphylococcus aureus* was sensitive for assessing TQ antibacterial activity. The MIC estimation of TQ could not be predicted according to the results of disc diffusion. The TQ has a profoundly negative impact on the norA efflux system that resulted in decreased *S. aureus* basal efflux pumping that was both naturally occurring and caused by CIP and DO. The anti-efflux action generated by TQ is similar to that seen with verapamil. The presence of TQ-MICs above the blood concentration maximum level requires further required investigations to improve TQ-pharmacokinetics, especially TQ-blood concentration maximum (C-max), to restore the activity of CIP and DO on the market. TQ ligand inhibited norA protein macromolecule and can therefore suppress the emergence of fluoroquinolone and doxycycline-resistant *S. aureus* and augment their antibacterial activities in clinical settings.

## Data Availability

All data generated or analyzed during this study are included in this published article and available on request.
